# A Rare Case of Transient Proximal Renal Tubular Acidosis in Pregnancy

**DOI:** 10.1155/2017/1342135

**Published:** 2017-11-29

**Authors:** Dennis Narcisse, Manyoo Agarwal, Aneel Kumar

**Affiliations:** ^1^University of Tennessee College of Medicine, Memphis, TN, USA; ^2^Department of Internal Medicine, University of Tennessee Health Science Center, Memphis, TN, USA

## Abstract

Renal tubular acidosis (RTA) is a disorder that has improper function of renal acid-base regulation and is rarely encountered during pregnancy. Currently, there is no clear evidence on management and outcomes in patients with this condition. We report a case of a previously healthy 23-year-old female at 30 weeks of gestation who presented with proximal RTA and had spontaneous resolution of the condition shortly after delivery.

## 1. Introduction

Renal tubular acidosis (RTA) is a group of disorders that are defined by defective renal acid-base regulation. The disease can be inherited, acquired, or idiopathic. RTA is classified into three major forms, which include distal (type 1), proximal (type 2), and hyperkalemic (type 4) forms. Distal RTA is characterized by decreased urinary acid secretion, proximal RTA has an impaired bicarbonate reabsorption, and hyperkalemic RTA is either an aldosterone deficiency or resistance [1].

RTA is rarely encountered during pregnancy and commonly has been reported with maternal substance abuse, such as toluene [2]. Less commonly, it is associated with other maternal diseases such as systemic lupus erythematous, chronic hepatitis, or diabetes [3]. RTA may also have an unclear etiology and sometimes can have a transient course with complete resolution after delivery [2].

We report a case of transient proximal RTA in a previously healthy 23-year-old patient at 30 weeks of gestation that spontaneously resolved after delivery.

## 2. Case

A 23-year-old previously healthy African American female, gravida 5 para 4, with no significant past medical history presented at 30 weeks of gestation with complaints of generalized weakness and fatigue for the previous two weeks. Review of systems was only significant for polyuria and polydipsia. Physical examination revealed normotensive blood pressure (122/66 mmHg), normal heart rate (72 beats per minute), and a fundus height that was consistent with 30 weeks of gestation. She had no other notable physical exam findings.

Labs revealed WBC 6.5 cells/uL, hemoglobin 13 g/dL, platelets 350, sodium 135 mmol/L, potassium 1.6 mmol/L, chloride 115 mmol/L, bicarbonate 14 mmol/l, BUN 2 mg/dL, creatinine 0.7 mg/dL, glucose 74 mg/dL, albumin 2.5 gm/dL, calcium 8.7 mg/dL, magnesium 2.1 mEq/L, and corrected serum anion gap of 10.55. Arterial blood gas revealed pH of 7.33, PCO_2_ 25 mmHg, PO_2_ 110 mmHg, and bicarbonate 13.2 moll/L. Urine studies were significant for urine pH of 5, urine anion gap of −1 (urine sodium 80 mmol/L, urine potassium 40 mmol/L, and urine chloride 121 mmol/L), and transtubular potassium gradient of 18 (serum osmolality 275 mosm/dL/L, urine osmolality 380 mosm/dL, urine potassium 40 mmol/L, and serum potassium 1.6 mmol/l), and both urine protein and glucose were negative.

The patient was subsequently diagnosed with type 2 (proximal) RTA based on the laboratory data ordered above. ANA, anti-SSA, anti-SSB, anti-dsDNA, anti-Smith, HIV, TSH, and hepatitis panel were all normal/nonreactive. During the hospital stay, she initially received a sodium bicarbonate drip totaling 300 mEq. This was eventually transitioned to potassium citrate 20 mEq orally three times per day. After one week, she underwent a spontaneous vaginal delivery of a premature fetus; however the baby died shortly after delivery secondary to respiratory failure. The patient was seen at a two-week follow-up visit in the outpatient clinic where serum potassium was 4.3 and serum bicarbonate was 27 mmol/L. Our patient did not require any further potassium or bicarbonate supplementation after pregnancy and her supplementation was then discontinued.

## 3. Discussion

The kidney has an important role in maintaining normal systemic acid base by controlling bicarbonate reabsorption and acid secretion. Loss of some tubular function can prevent the kidney from maintaining this balance [4]. The group of diseases characterized as renal tubular acidosis (RTA) refers to a condition in which there is normal serum anion gap or hyperchloremic metabolic acidosis in the presence of well-preserved glomerular function. This is caused by the inability of the renal tubule to reabsorb bicarbonate or to secrete hydrogen ions [5]. In most sources, primary RTA is divided into a proximal (type II), distal (type I), and a hyperkalemic form associated with hyperaldosteronism or aldosterone resistance (Type IV).

Distal RTA (type 1) is the most common form of primary RTA in most Western countries. Distal RTA occurs due to the inability of the renal tubules to decrease urine pH and increase urinary ammonium excretion during sustained hyperchloremic metabolic acidosis and hypokalemia [5]. This is caused by a failure to reabsorb bicarbonate by the intercalated cells in the collecting duct [6]. The disease can be inherited or more commonly it can be a complication of other systemic diseases. Distal RTA can also lead to increased loss of urinary calcium resulting in osteopenia, osteomalacia, nephrocalcinosis, and even secondary hyperparathyroidism [2]. Patients with distal RTA typically present with signs and symptoms related to severe hypokalemia such as proximal muscle weakness, polydipsia, and polyuria. Treatment of these patients typically include supplementing with sodium or potassium bicarbonate [6].

Proximal RTA (type 2) is associated with a decreased ability of the proximal nephron to reabsorb bicarbonate. Patients with proximal RTA can be asymptomatic or as in our case present with weakness and paralysis secondary to severe hypokalemia. The hyperchloremic metabolic acidosis in proximal RTA is usually less severe because the tubular system is still capable of retaining bicarbonate distally [6]. This type of RTA is most commonly an inherited condition associated with Fanconi's syndrome, in which there is also a tubular loss of glucose, calcium, phosphate, and amino acids [2].

There is a further subdivision of proximal RTA, which is a rare condition called isolated proximal RTA. This form can either be classified as an autosomal dominant, autosomal recessive, or sporadic isolated proximal RTA. In either the autosomal dominant or recessive form, the RTA usually is a lifelong condition after presentation. However, the sporadic isolated proximal RTA is typically a sporadic event that resolves sometime after treatment [2]. Our case is very likely a presentation of the sporadic isolated proximal RTA because of the spontaneous resolution shortly after treatment and delivery of the fetus.

Pregnancy is reported to worsen either type of RTA. In pregnancy, there is an understood mild physiological respiratory alkalosis and a hyperfiltration in the kidneys secondary to increased blood volume. This hyperfiltration results in an increased loss of some electrolytes leading to a higher requirement of potassium and bicarbonate [3]. Some forms of RTA can cause extreme hypokalemia due to these factors. Both distal and proximal RTA can cause hypokalemia which usually is a result of the renal system attempting to compensate for the metabolic acidosis by excreting potassium [2]. Rhabdomyolysis, commonly caused by severe hypokalemia, is a potentially life threating syndrome that results from the breakdown of muscle fibers that leak into circulation. In pregnancy, there is an increased incidence of hypokalemia induced rhabdomyolysis as seen in a case report by two of the cases in Table 1. In both cases of rhabdomyolysis, the patients presented with muscle pain and severe cramps. Electrolyte abnormalities, renal function, and liver enzymes all need to be monitored and corrected quickly especially in pregnant patients to prevent adverse outcomes [7, 8].

There are some case reports of RTA presenting for the first time in pregnant patients as seen in Table 1. In the medical literature, RTA in pregnancy could be secondary to a maternal systemic disease or ingestion of medications like Dyazide for hypertension during pregnancy or following toluene abuse. Some systemic disorders that have been reported to cause RTA were diabetic ketoacidosis, thyrotoxicosis, alcoholic liver disease, and sickle cell disease. However, in the five cases that were found, only one had a clear cause of RTA. In a report by Mallett et al., the patient had a history of ibuprofen and codeine abusing for the previous six years. The cause of the distal RTA in this case appears clearly linked to her substance abuse, and her renal function and potassium requirements significantly improved after stopping ibuprofen and codeine [9]. In the case by Firmin et al., the previously healthy patient presented with muscle weakness and body pains and was diagnosed with sporadic isolated proximal RTA. The patient responded well to treatment and delivered a healthy infant. Her RTA persisted in a very mild form at her follow-up visits [2]. In the last case, Seoud et al. reported on a previously healthy patient who presented with no signs or symptoms of hypokalemia, but she was incidentally diagnosed with RTA on labs drawn for possible preterm labor. After treatment and delivery of a healthy infant, she gradually had complete resolution of her acidosis and electrolyte abnormalities over the course of six months [3]. In all of the cases found in Table 1, the acidosis did not have any major effect on the fetus.

Previous reports of RTA in pregnancy, however, have offered little discussion on the effects of RTA on fetal development [10]. Severe acute metabolic acidosis in pregnancy has been suggested to cause decreased fetal circulation leading to possible fetal distress or demise [8]. In our case, the patient went into spontaneous premature delivery of the fetus that did not survive. The effects of RTA on fetal outcome are unclear. RTA in pregnancy and the negative effects can be corrected, as seen in Firmin et al. work, whenever the condition is diagnosed early and permanent damage has not yet occurred [2]. However, there have been cases, such as that of Jain et al., where RTA was found to be a cause of multiple loss of pregnancies in a 30-year-old patient [11].

Also, in our case, the patient had no previous past medical history and spontaneously began having hypokalemia symptoms from her sporadic RTA. The patient did not have any evident causes of her disease and did not report any family history or prior complications with previous pregnancies. Seoud et al. reported their patient with proximal RTA initially presented with preterm labor. Their case also resulted in resolution of electrolyte abnormalities in less than a year after delivery [3]. On our patient's labs, there was a very evident hypokalemic hyperchloremic metabolic acidosis, urine studies were consistent with poor compensation by the tubular system, and urinary pH was less than 5.3 with a low serum bicarbonate. The patient had significantly low albumin levels which can cause a reduction in unmeasured anions. For this reason, a corrected anion gap was still collected and it was found to be normal gap of 10.55. These factors are diagnostic for proximal RTA [6]. Treatment goal for proximal RTA is to increase the serum concentration of bicarbonate to as close to normal as possible.

The patient had no systemic disorders and her electrolyte abnormalities quickly resolved after delivery of the preterm fetus. This leads us to believe that our case was isolated sporadic proximal RTA induced during pregnancy. Unfortunately, our patient presented several weeks after being symptomatic, which led to delayed acidosis correction and a preterm delivery. The newborn had respiratory failure and died shortly after birth. It is unclear if earlier treatment would have prevented this outcome.

## 4. Conclusion

In conclusion, we presented a very rare case of isolated sporadic proximal RTA (type 2) in a previously healthy pregnant patient. She has no past medical history and no clear inciting causes of her presenting condition. She responded well to treatment and one week later delivered a preterm infant who did not survive. Shortly after her delivery, she spontaneously and completely resolved her condition no longer needing any treatment for RTA.

## Figures and Tables

**Table 1:** Reported cases and outcomes of renal tubular acidosis (RTA) that presented during pregnancy [2, 3, 7–9].

Case	Year	Type of RTA	Outcome
Srisuttayasathien	2015	Distal RTA	Rhabdomyolysis induced by hypokalemia from distal RTA; successful delivery and being healthy on follow-up.

Mallett et al.	2011	Distal RTA (ibuprofen related)	Distal RTA induced by ibuprofen and codeine abuse that resolved after delivery of healthy baby at 37 weeks. Some renal dysfunction persisted on follow-up.

Muthukrishnan et al.	2010	Distal RTA	Persistent hypokalemia leading to rhabdomyolysis; successful delivery and complete resolution of symptoms.

Firmin et al.	2007	Proximal RTA	Delivery of a healthy infant; mild persistence of hypokalemia at 1-year follow-up.

Seoud et al.	2000	Transient RTA	Delivery of a healthy infant; complete resolution of symptoms at follow-up.
